# Comparison of ceftobiprole 5 μg disk diffusion, MIC test strip, and broth microdilution for susceptibility testing of *Staphylococcus aureus* clinical isolates

**DOI:** 10.1128/jcm.00125-26

**Published:** 2026-03-23

**Authors:** Xinying Wang, Yujing Tian, Yan Jin, Shujing Sui, Zhijun Zhang

**Affiliations:** 1 Department of Laboratory Medicine, The Affiliated Taian City Central Hospital of Qingdao University, Taian, China; 2 Shandong Provincial Key Medical and Health Laboratory of Anti-Drug Resistant Drug Research, The Affiliated Taian City Central Hospital of Qingdao University, Taian, China; 3 Department of Laboratory Medicine, Shandong Provincial Hospital Affiliated to Shandong First Medical University, Shandong Provincial Hospital, Jinan, China; 4 Department of Gastroenterology, The Affiliated Taian City Central Hospital of Qingdao University, Taian, China

**Keywords:** ceftobiprole, *Staphylococcus aureus*, MRSA, broth microdilution, MIC Test Strip, disk diffusion, methodology

## Abstract

Ceftobiprole is a new-generation cephalosporin with activity against *Staphylococcus aureus,* but a comprehensive evaluation of MIC Test Strip (MTS) and disk diffusion (DD) methods using EUCAST and FDA breakpoint is lacking. Using broth microdilution (BMD) as the reference standard, we assessed the performance of MTS and DD for determining ceftobiprole susceptibility among 422 clinical *S. aureus* isolates. Ceftobiprole retained potent activity, with MIC₅₀/MIC₉₀ of 0.5/1 mg/L and an overall susceptibility rate of 98.8%; methicillin-resistant *Staphylococcus aureus* (MRSA) showed 97.6% susceptibility. MTS demonstrated high agreement with BMD, achieving 98.8% categorical agreement (CA), 1.2% major error (ME), and zero very major error (VME) under European Committee on Antimicrobial Susceptibility Testing (EUCAST) criteria, meeting the acceptability limits of ISO 20776-2:2021 and CLSI M52. DD performance was breakpoint-dependent: under EUCAST criteria, the overall CA was 89.6%, with 10.6% ME and 0 VME. The 40.3% of MRSA that fell into the EUCAST-defined Area of Technical Uncertainty (ATU) zone were all shown to be susceptible by the reference BMD method. Under Food and Drug Administration (FDA) breakpoints, the CA rose to 94.1% and ME fell to 1.7%. Consequently, MTS offers a reliable routine option; DD is suitable for routine testing under FDA breakpoints, while for EUCAST users, MRSA isolates in the ATU zone should be confirmed by BMD or MTS. VME was not determined under FDA breakpoints due to the absence of resistant isolates.

**IMPORTANCE:**

Ceftobiprole offers a β-lactam option for *Staphylococcus aureus*, including methicillin-resistant *Staphylococcus aureus* (MRSA); however, its susceptibility testing remains insufficiently examined across both European Committee on Antimicrobial Susceptibility Testing (EUCAST) and Food and Drug Administration (FDA) interpretive criteria. We systematically compared MIC Test Strip (MTS) and disk diffusion (DD) with broth microdilution (BMD), demonstrating breakpoint-specific performance for methicillin-susceptible *Staphylococcus aureus* (MSSA) and MRSA and providing practical recommendations that allow laboratories to report accurate results regardless of the guidelines they follow.

## INTRODUCTION

Methicillin-resistant *Staphylococcus aureus* (MRSA) is a leading cause of healthcare-associated infections globally, posing a major therapeutic challenge due to its high virulence and multidrug resistance (1, 2). According to the 2024 CHINET national surveillance report, MRSA accounts for 32.3% of all *Staphylococcus aureus* clinical isolates from 78 hospitals across China, including 72 tertiary-care and 6 secondary-care institutions (3). MRSA harbors the *mecA* (or rarely *mecC*) gene encoding penicillin-binding protein PBP2a, conferring resistance to most β-lactams (4); separately, vancomycin-intermediate (VISA), vancomycin-resistant (VRSA), and linezolid-resistant MRSA isolates have been reported worldwide (5–7), narrowing conventional treatment options.

Ceftobiprole is a new-generation parenteral cephalosporin that acylates and inactivates bacterial penicillin-binding proteins (PBPs), including PBP2a in *S. aureus*, thereby preventing peptidoglycan cross-linking and ultimately causing osmotic lysis. It is one of the few β-lactams that retain potent activity against MRSA, VISA, VRSA, and most common gram-negative bacilli (8, 9). Beyond China and the USA, ceftobiprole has achieved global regulatory approval and clinical availability in multiple regions over the past decade. It was first launched in Canada and Switzerland in 2008 under the trade names Zeftera and Zevtera, respectively, initially indicated for complicated skin and soft-tissue infections (10). In the USA, the Food and Drug Administration (FDA) granted approval on 3 April 2024, expanding its indications to include *S. aureus* bacteremia (including right-sided infective endocarditis) and other serious infections, with clinical availability commencing in May 2025 (11). In China, it received National Medical Products Administration (NMPA) approval in October 2020 (trade name: Saibipu) for adult community-acquired and hospital-acquired pneumonia and has since been accessible in tertiary care hospitals nationwide, serving as a key therapeutic option for MRSA-related infections (12). Its progressive global availability, particularly in major markets like China and the USA, underscores the growing clinical demand for reliable susceptibility testing methods to guide optimal use of this agent. The global SENTRY antimicrobial surveillance program reported MIC_50_/MIC_90_ values of 0.5/2 mg/L for ceftobiprole against 2,930 *S. aureus* isolates, with a susceptibility rate of 99.7% (13). Multicenter data from China revealed almost identical MIC_50_/MIC_90_ values of 1/2 mg/L against MRSA and >99% susceptibility (14, 15). Consequently, ceftobiprole is now recommended as an option by national and international guidelines for the treatment of hospital- and community-acquired pneumonia, as well as skin and soft-tissue infections caused by gram-positive pathogens including MRSA (16, 17). The agent does not require routine therapeutic drug monitoring and can be used without dose adjustment in renal impairment, elderly patients, and children, expanding its clinical utility (18).

At present, four phenotypic methods are available for ceftobiprole susceptibility testing: disk diffusion (DD), MIC Test Strip (MTS), broth microdilution (BMD), and automated systems. DD and MTS are inexpensive, offer flexible single-agent testing without the need for dedicated commercial panels, and have already been adopted by most clinical microbiology laboratories in China. BMD is recognized as the reference procedure by both Clinical and Laboratory Standards Institute (CLSI) and European Committee on Antimicrobial Susceptibility Testing (EUCAST), but its labor-intensive protocol and stringent technical demands limit routine use. Although automated platforms offer high throughput, ceftobiprole is not yet included in most commercial antibiotic susceptibility testing (AST) panels. Notably, the ThermoFisher SensiTitre platform is currently the only commercial automated system with FDA-cleared ceftobiprole susceptibility testing capability via its dedicated AST panel (19). However, this ceftobiprole-specific testing panel has not been made available or clinically applied in China.

Compounding the challenges of method selection, discrepancies in ceftobiprole susceptibility interpretation standards exist among different authoritative bodies. Specifically, the EUCAST and the FDA have proposed distinct breakpoints, while the CLSI has not yet established official breakpoints. This dual gap of limited standardized methods and inconsistent interpretation criteria highlights the need for systematic evaluation of ceftobiprole susceptibility testing methodologies based on unified criteria. Large-scale, head-to-head comparisons of these methodologies remain scarce. We, therefore, undertook the present study to evaluate the accuracy and reproducibility of MTS and DD against those of BMD for ceftobiprole susceptibility testing of 422 recent clinical *S. aureus* isolates. By concurrently applying FDA and EUCAST interpretive criteria, we compared the performance characteristics of the two methods, aiming to provide an evidence-based reference for method selection, breakpoint interpretation, and clinical decision-making.

## MATERIALS AND METHODS

### Bacterial isolates

A total of 422 nonduplicate *S. aureus* clinical isolates were collected from December 2023 to December 2024 at the Affiliated Tai’an Central Hospital of Qingdao University. Only the first isolate per patient per anatomical site was retained. The collection comprised 256 methicillin-susceptible *S. aureus* (MSSA; 60.7%) and 166 MRSA (39.3%). Specimen distribution was as follows: wound and soft-tissue exudates 210/422 (49.8%), sputum 105/422 (24.9%), sterile body fluids (pleural, ascitic, synovial, or drainage fluids) 50/422 (11.8%), blood cultures 41/422 (9.7%), and miscellaneous sources 16/422 (3.8%). All isolates were identified to the species level using matrix-assisted laser desorption/ionization time-of-flight mass spectrometry (Autof ms1000, Autobio, China). After verification, pure cultures were suspended in brain–heart infusion broth containing 15% (vol/vol) glycerol and stored at −80°C until tested. Prior to each susceptibility assay, isolates were subcultured twice on sheep-blood agar to ensure purity and viability. MRSA was defined as *S. aureus* with either oxacillin MIC > 2 mg/L or cefoxitin disk zone < 22 mm, according to the 2025 EUCAST breakpoints (20).

### Antimicrobial susceptibility testing

Ceftobiprole MTS and disks were obtained from Liofilchem (Roseto degli Abruzzi, Italy); the strip range was 0.002–32 mg/L, and each disk contained 5 μg ceftobiprole. Ceftobiprole powder (lot no. ZP-230712; Zhenzhun Biotech, Shanghai, China) was used to prepare cation-adjusted Mueller-Hinton broth (CAMHB; BD, USA) doubling-dilution panels (0.015–32 mg/L) according to ISO 20776-1:2019 (21). The CAMHB was bought from the Becton, Dickinson and Company (USA). A 0.5 McFarland suspension (approximately 1 × 10⁸ CFU/mL) was prepared and diluted 1:100 in CAMHB to approximately 1 × 10⁶ CFU/mL. Fifty microliters of this diluted inoculum was added to each 50 µL drug-containing well, yielding a final concentration of approximately 5 × 10^5^ CFU/mL. Panels were incubated at 35 ± 1°C in ambient air for 16–20 h before reading. All three methods were inoculated from the same 0.5 McFarland suspension within 15 min. DD and MTS testing were performed on the same Mueller–Hinton agar plate (MH; Zhengzhou, China) following EUCAST Disk Diffusion Method for Antimicrobial Susceptibility Testing (22) and the manufacturer’s instructions. Plates were incubated at 35 ± 1°C in ambient air for 16–20 h. The MIC value by MTS was defined as the lowest concentration at which the elliptical inhibition zone intersected the scale, corresponding to 100% growth inhibition. Results were read independently by two technologists blinded to each other’s findings; discrepancies were resolved by a third reader, and the concordant value was retained. When an agreement could not be reached, the test was repeated. For susceptibility interpretation, the primary reference was the 2025 EUCAST Breakpoint tables (Version 15.0) (20). For comparative analysis to evaluate the impact of different breakpoint systems, interpretations were also performed according to the FDA breakpoints for ceftobiprole against *S. aureus*. Breakpoint criteria and quality control specifications for both standards are detailed in Table 1 (23, 24). All quality control (QC) runs yielded results within specified ranges, meeting both standards.

**TABLE 1:** Breakpoint interpretations applied to ceftobiprole antimicrobial susceptibility testing results^*a*^

Standard	Method	S	I	R	ATU	QC strain	QC range
EUCAST	MIC (mg/L)	≤2^*b*^	–^*c*^	＞2^*b*^	2	ATCC29213	0.12–1
	DD (mm)	≥17^*b*^	–	＜17^*b*^	16–17	ATCC29213	22–28
FDA	MIC (mg/L)	≤2	4	≥8	–	ATCC29213	0.12–1
	DD (mm)	≥16	14–15	≤13	–	ATCC25923	20–27

^*a*^ FDA, U.S. Food and Drug Administration; EUCAST, European Committee on Antimicrobial Susceptibility Testing; S, susceptible; I, intermediate; R, resistant. ATU, Area of Technical Uncertainty, applies to MRSA only; QC, quality control.

^*b*^ Methicillin-susceptible isolates can be reported susceptible to ceftobiprole without further testing.

^*c*^ “–” signifies the absence of an intermediate (I) class.

### Statistical analysis

Using BMD as the reference, the performance of MTS and DD was evaluated with the dBETS software according to DePalma G et al’s studies (25). The evaluation indicators for the method refer to the guidelines in ISO 20776-2:2021 (26), including the categorical agreement (CA), major error (ME), and very major error (VME). And if CA ≥ 90%, ME ≤ 3%, and VME ≤ 3%, the assessment method is acceptable. For FDA breakpoints CA, essential agreement (EA), VME, ME, and minor error (mE) were accepted: CA ≥ 90%, EA ≥ 90%, ME ≤ 3%, VME ≤ 1.5%, and mE < 10% based on CLSI M52 (27).

## RESULTS

### Ceftobiprole broth microdilution

Ceftobiprole exhibited MICs of ≤0.015–4 mg/L against the 422 *S. aureus* isolates, with MIC₅₀ and MIC₉₀ values of 0.5 mg/L and 1 mg/L, respectively; the overall susceptibility rate was 98.8% (417/422). Among the 256 MSSA strains, 99.6% (255/256) were susceptible, displaying MIC₅₀/₉₀ of 0.25/0.5 mg/L, whereas the 166 MRSA isolates showed 97.6% susceptibility (162/166) and MIC₅₀/₉₀ of 1/2 mg/L (Table 2).

**TABLE 2:** MIC distribution of ceftobiprole against *S. aureus* (BMD, mg/L)^*a*^

	No. of isolates with the ceftobiprole MIC (mg/L)														
Organism	≤0.015	0.03	0.06	0.125	0.25	0.5	1	2	4	8	16	≥32	Total	MIC_50_	MIC_90_
*S. aureus*	2	0	0	11	168	137	61	38	5	0	0	0	422	0.5	1
MSSA	1	0	0	11	161	73	7	2	1	0	0	0	256	0.25	0.5
MRSA	1	0	0	0	7	64	54	36	4	0	0	0	166	1	2

^*a*^ MIC, minimum inhibitory concentration; BMD, broth microdilution; MIC_50_, MIC for inhibiting 50% of the isolates; MIC_90_, MIC for inhibiting 90% of the isolates; MSSA, methicillin (oxacillin)-susceptible *S. aureus;* MRSA, methicillin-resistant *S. aureus*.

### Ceftobiprole MIC Test Strip

**Fig 1:**
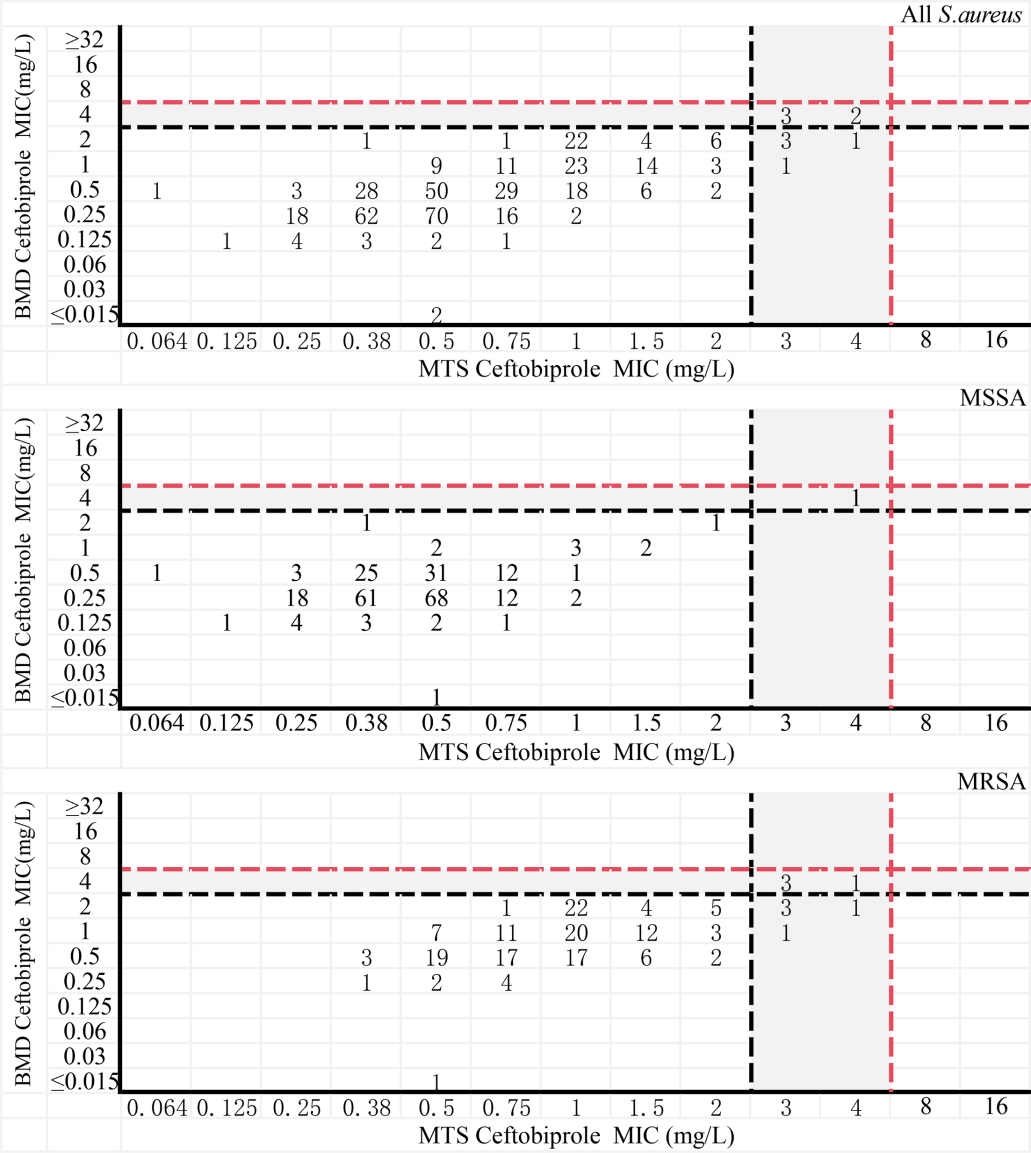
Scatter plot comparing ceftobiprole MICs determined by MTS with reference BMD for 422 *S. aureus* isolates. Black dashed lines: EUCAST breakpoints (MIC ≤ 2 mg/L = susceptible, MIC > 2 mg/L = resistant); red dashed lines: FDA breakpoints (MIC ≤ 2 mg/L = S, MIC = 4 mg/L = I, MIC ≥ 8 mg/L = R). Note: The susceptible breakpoint is identical for both standards. The gray shaded area denotes the FDA intermediate category only.

Ceftobiprole MICs ranged from 0.064 to 4 mg/L, with MIC₅₀ and MIC₉₀ values of 0.5 mg/L and 1 mg/L, respectively, yielding an overall susceptibility rate of 97.6% (412/422). When using EUCAST breakpoints, compared with BMD, MTS achieved a CA of 98.8% (417/422), EA of 91.0% (384/422), ME of 1.2% (5/417), and VME of 0 (0/5). Among the 256 MSSA isolates, CA was 100.0% (256/256), EA 91.0% (233/256), ME 0 (0/255), and VME 0 (0/1); for the 166 MRSA isolates, CA was 97.0% (161/166), EA 91.0% (151/166), ME 3.0% (5/162), and VME 0 (0/4). Under the FDA standard, the performance of the MTS method in terms of CA and EA was consistent with that under the EUCAST standard across all *S. aureus* subgroups, though VME could not be determined due to the absence of ceftobiprole-resistant *S. aureus*. The only additional metric assessed by the FDA standard is mE, for which the overall mE of MTS for *S. aureus* was 1.2% (5/422), with 0 in MSSA and 3.0% (5/166) in MRSA. (Table 3; Fig. 1)

**TABLE 3:** Concordance of different methods for detecting ceftobiprole susceptibility in *S. aureus^a^*

Organism	Methods	Parameter	*S. aureus*	MSSA	MRSA
EUCAST	MTS	ME	5/417 (1.2%)	0/255 (0)	5/162 (3.0%)
		VME	0/5 (0)	0/1 (0)	0/4 (0)
		EA	384/422 (91.0%)	233/256 (91.0%)	151/166 (91.0%)
		CA	417/422 (98.8%)	256/256 (100.0%)	161/166 (97.0%)
	DD	ME	44/417 (10.6%)	1/255 (0.4%)	43/162 (26.5%)
		VME	0/5 (0)	0/1 (0)	0/4 (0)
		CA	378/422 (89.6%)	255/256 (99.6%)	123/166 (74.1%)
FDA	MTS	ME	0/417 (0)	0/255 (0)	0/162 (0)
		mE	5/422 (1.2%)	0/256 (0)	5/166 (3.0%)
		VME	NA	NA	NA
		EA	384/422 (91.0%)	233/256 (91.0%)	151/166 (91.0%)
		CA	417/422 (98.8%)	256/256 (100%)	161/166 (97.0%)
	DD	ME	7/417 (1.7%)	0/255 (0)	7/162 (4.3%)
		mE	18/422 (4.3%)	2/256 (0.8%)	16/166 (9.6%)
		VME	NA	NA	NA
		CA	397/422 (94.1%)	254/256 (99.2%)	143/166 (86.1%)

^*a*^ FDA, U.S. Food and Drug Administration; EUCAST, European Committee on Antimicrobial Susceptibility Testing; ME, major error; mE, minor error; VME, very major error; MTS, MIC Test Strip; DD, disk diffusion; MSSA, methicillin (oxacillin)-susceptible *S. aureus;* MRSA, methicillin-resistant *S. aureus;* NA*,* no resistant isolates available for VME calculation.

### Ceftobiprole disk diffusion

**Fig 2:**
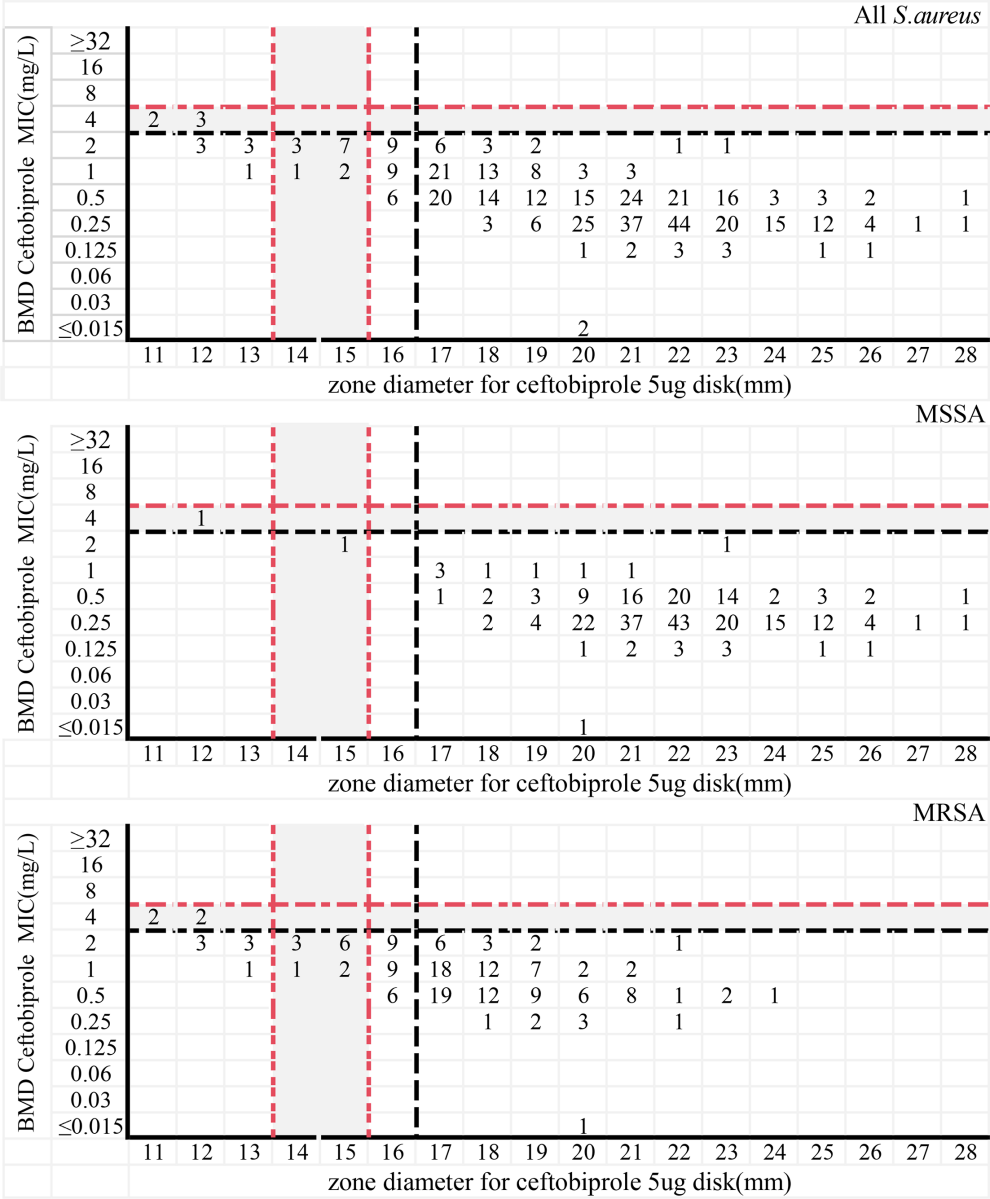
Scatter plot comparing the MIC values of ceftobiprole determined by the inhibition zone diameters of disk diffusion with the results of reference BMD when testing *S. aureus*. Black dashed line: EUCAST breakpoint (S ≥ 17 mm, R < 17 mm); red dashed line: FDA breakpoint (S ≥ 16 mm, I 14–15 mm, and R ≤ 13 mm). The gray band denotes the FDA intermediate zone (14–15 mm).

Ceftobiprole disk diffusion zone diameters ranged from 11 to 28 mm. Using EUCAST breakpoints, the CA with BMD was 89.6% (378/422), with a ME rate of 10.6% (44/417) and VME was 0 (0/5). Among the 256 MSSA isolates, CA reached 99.6% (255/256), ME was 0.4% (1/255), and VME remained 0 (0/1). In contrast, the 166 MRSA isolates showed a CA of only 74.1% (123/166) and an ME of 26.5% (43/162), while VME was still 0 (0/4). Under the FDA breakpoints, the DD method exhibited improved performance compared with that under the EUCAST standard, though suboptimal results were still observed in MRSA isolates. Specifically, the overall CA of DD with BMD increased to 94.1% (397/422), and the ME rate decreased to 1.7% (7/417). For MSSA isolates, CA was 99.2% (254/256) and ME was 0 (0/255). For MRSA isolates, CA improved to 86.1% (143/166) and ME decreased to 4.3% (7/162). Consistent with the FDA assessment criteria, mE was additionally evaluated for DD: the overall mE was 4.3% (18/422), with 0.8% (2/256) in MSSA isolates and 9.6% (16/166) in MRSA isolates. Notably, VME was not applicable under FDA breakpoints due to the absence of resistant isolates. (Table 3; Fig. 2)

### ATU zone analysis

In this study, 10 MRSA isolates fell into the MTS ATU zone (MIC = 2 mg/L); all were confirmed susceptible by BMD. For disk diffusion, 40.3% (67/166) of MRSA strains produced zones within the ATU range (16–17 mm): 24 isolates gave a 16-mm zone and 43 a 17-mm zone. Every one of these isolates was also confirmed susceptible by BMD, with MICs ranging from 0.5 to 2 mg/L (Tables S1 and S2). Under FDA breakpoints, the same MIC = 2 mg/L or zone ≥ 16 mm would be classified as susceptible and MIC = 4 mg/L or zone 14–15 mm would be intermediate.

## DISCUSSION

MRSA remains a major healthcare-associated pathogen globally, with an approximately 30% isolation rate among *S. aureus* in China (3, 28). Its limited treatment options highlight the clinical value of ceftobiprole, which retains potent *in vitro* activity against MRSA via PBP2a targeting (10), supporting the need for reliable susceptibility testing methods evaluated in this study. Clinical data demonstrate that ceftobiprole is non-inferior to daptomycin for complicated *S. aureus* bacteremia and achieves therapeutic concentrations in cerebrospinal fluid (29). The TARGET study further showed that ceftobiprole was superior to vancomycin for *S. aureus* bloodstream infection (30). Consequently, ceftobiprole represents a valuable new option for managing infections caused by multidrug-resistant staphylococci.

Using BMD, the reference method endorsed by both EUCAST and FDA, this study confirmed that ceftobiprole retains exceptional activity against the 422 contemporary *S. aureus* isolates. These values are essentially concordant with the global SENTRY program (13, 31) and with recent multicenter data from China (14, 15). Only 4 MRSA isolates exhibited an MIC of 4 mg/L, indicating that the local epidemic MRSA population remains susceptible to ceftobiprole. Consequently, the agent represents a reliable empirical or targeted option for severe pneumonia, complicated skin and soft-tissue infections, and bloodstream infections caused by MRSA.

For MTS, the method exhibited high concordance with the reference BMD assay across both breakpoint systems. Specifically, MTS achieved an overall CA of 98.8% and EA of 91.0%, with an ME rate of 1.2% and 0 VMEs under EUCAST criteria; VME was not applicable under FDA breakpoints due to the absence of resistant isolates. Stratified analysis by strain type revealed that MTS performed perfectly for MSSA, yielding 100% CA and no errors under both standards. For MRSA, MTS maintained a CA of 97.0% and ME rates of less than 3.0% under both EUCAST and FDA frameworks. Notably, all 5 ME cases occurred at an MIC of 4 mg/L, which exceeds the EUCAST breakpoint and is therefore categorized as resistant, whereas the FDA breakpoint assigns it to intermediate. This confirms that the observed MEs stem from inherent differences in the two interpretive criteria rather than technical limitations of the MTS method itself.

In contrast, DD demonstrated marked performance discrepancies between the two breakpoint systems. Under EUCAST criteria, DD showed suboptimal overall performance, with an overall CA of 89.6% and a high ME rate of 10.6%; this poor performance was primarily driven by MRSA isolates, which exhibited a low CA of 74.1% and an ME rate as high as 26.5%. However, when interpreted using FDA breakpoints, DD performance improved substantially: overall CA increased to 94.1%, and the MRSA-associated ME rate dropped sharply to 4.3%. Stratified analysis further showed that DD had a low ME rate of 0.4% (EUCAST) and 0 (FDA) for MSSA, indicating its reliability for MSSA testing regardless of the standard employed. Under EUCAST criteria, no VME was detected (0/5); under FDA breakpoints, VME could not be determined due to the absence of resistant isolates.

Detailed analysis revealed that 40.3% of MRSA isolates fell into the DD ATU range, as defined by EUCAST 2025 breakpoints (20), yet all these isolates were confirmed as susceptible by the reference BMD method. This discordance directly explains the suboptimal performance of DD under EUCAST criteria—specifically, the high ME rate of 26.5% for MRSA—whereby DD tends to misclassify ATU-range isolates as non-susceptible. In stark contrast, when interpreted using FDA breakpoints, the same 16–17 mm inhibition zone diameter is categorized as susceptible, which aligns with BMD results and accounts for the marked reduction in the DD-associated MRSA ME rate to 4.3%. Such misclassification under EUCAST criteria carries tangible clinical implications as it may prompt unnecessary dose escalation or the empirical use of broader-spectrum alternative agents, thereby increasing the risk of collateral resistance development. We therefore recommend that DD be reserved for preliminary MRSA screening; any isolate whose inhibition zone falls within the EUCAST-defined ATU should be reflex-tested by MTS or BMD to ensure accurate susceptibility categorization. For laboratories adopting FDA breakpoints, DD may serve as a more reliable testing method for MRSA given its alignment with BMD results in the 16–17 mm range.

This study has several limitations. First, isolates were collected from a single tertiary care hospital in Tai’an, limiting the generalizability of findings to regions with divergent *S. aureus* epidemiological profiles. Second, no targeted characterization was performed on the five identified ceftobiprole-non-susceptible isolates, especially non-susceptible MSSA, precluding the exploration of their unique genetic/phenotypic traits and associated resistance mechanisms. Future multicenter surveillance integrating molecular assays is needed to confirm the stability of ceftobiprole susceptibility and diagnostic method reliability, as well as to elucidate the mechanistic basis of reduced susceptibility in atypical *S. aureus* strains.

### Conclusion

In summary, ceftobiprole exhibited potent *in vitro* activity against all 422 clinical *S. aureus* isolates, with 97.6% of MRSA susceptible under both 2025 EUCAST and FDA breakpoints, supporting its potential as an empirical first-line β-lactam for MRSA-associated infections. MTS showed excellent concordance with reference broth BMD across both standards, making it ideal for routine testing in resource-limited laboratories. DD performed reliably for MSSA in both frameworks. For MRSA, DD achieved high accuracy under FDA criteria (CA 94.1% and ME 1.7%), making it suitable for routine testing; however, under EUCAST criteria, the accuracy was lower (CA 74.1%) due to the high proportion of isolates falling in the ATU zone (16–17 mm), which required MTS/BMD confirmation to avoid misclassification. Collectively, our data guide laboratories in selecting susceptibility testing methods based on their preferred breakpoint standard.

## Data Availability

All data generated or analyzed during this study are included in this article.

## SUPPLEMENTAL MATERIAL

Table S1 jcm.00125-26-s0001.doc Correlation among disk diffusion, MIC test strip, and BMD for MRSA isolates with ceftobiprole zone diameters of 16–17 mm.

Table S2 jcm.00125-26-s0002.doc Correlation among disk diffusion, MIC test strip, and BMD for MRSA isolates with ceftobiprole MTS of 2 mg/L.

ASM does not own the copyrights to Supplemental Material that may be linked to, or accessed through, an article. The authors have granted ASM a non-exclusive, world-wide license to publish the Supplemental Material files. Please contact the corresponding author directly for reuse.
