# Supplementary material for: Comparison of ceftobiprole 5 μg disk diffusion, MIC test strip, and broth microdilution for susceptibility testing of Staphylococcus aureus clinical isolates
Source: J Clin Microbiol. 2026 Mar 23;64(4):e00125-26. doi: 10.1128/jcm.00125-26 (PMC13059733; doi:10.1128/jcm.00125-26)
Supplement: Table S1 — Correlation among disk diffusion, MIC test strip, and BMD for MRSA isolates with ceftobiprole zone diameters of 16–17 mm. [file jcm.00125-26-s0001.doc]

Table S1. The correlation among disk diffusion, MTS, and BMD for MRSA with ceftobiprole zones of 16 to 17 mm

| Strain | BMD (mg/L) | MTS (mg/L) | DD (mm) |
| --- | --- | --- | --- |
| 408 | 0.5 | 2 | 17 |
| 461 | 0.5 | 1.5 | 16 |
| 462 | 1 | 2 | 17 |
| 463 | 0.5 | 1.5 | 16 |
| 470 | 0.5 | 0.5 | 16 |
| 471 | 0.5 | 0.5 | 17 |
| 472 | 0.5 | 0.5 | 17 |
| 484 | 0.5 | 1 | 17 |
| 491 | 1 | 1.5 | 16 |
| 499 | 0.5 | 1 | 16 |
| 505 | 1 | 1 | 16 |
| 507 | 2 | 1 | 17 |
| 509 | 0.5 | 0.5 | 17 |
| 510 | 0.5 | 0.75 | 17 |
| 513 | 1 | 1 | 17 |
| 514 | 0.5 | 1 | 16 |
| 518 | 1 | 0.75 | 17 |
| 519 | 1 | 1.5 | 16 |
| 523 | 1 | 0.75 | 17 |
| 535 | 1 | 0.75 | 17 |
| 538 | 2 | 1 | 17 |
| 539 | 1 | 1 | 16 |
| 541 | 1 | 1 | 16 |
| T1 | 0.5 | 1 | 17 |
| T3 | 0.5 | 1 | 17 |
| T25 | 0.5 | 1 | 17 |
| T35 | 0.5 | 1.5 | 17 |
| T37 | 0.5 | 1.5 | 17 |
| T42 | 0.5 | 2 | 17 |
| T47 | 0.5 | 1 | 17 |
| T53 | 1 | 1.5 | 17 |
| T60 | 0.5 | 0.75 | 17 |
| T72 | 1 | 1 | 17 |
| T79 | 1 | 1 | 17 |
| T89 | 1 | 1.5 | 17 |
| T90 | 1 | 1.5 | 17 |
| T101 | 1 | 1.5 | 17 |
| T113 | 1 | 1.5 | 16 |
| T130 | 0.5 | 1 | 16 |
| T148 | 0.5 | 0.5 | 17 |
| T150 | 0.5 | 1 | 17 |
| T156 | 0.5 | 0.5 | 17 |
| T205 | 1 | 1 | 17 |
| Z1 | 2 | 1 | 16 |
| Z2 | 2 | 1 | 16 |
| Z3 | 2 | 1.5 | 17 |
| Z4 | 2 | 1 | 16 |
| Z5 | 2 | 1 | 17 |
| Z6 | 2 | 1 | 17 |
| Z7 | 2 | 1 | 17 |
| Z9 | 1 | 1 | 16 |
| Z10 | 1 | 0.75 | 17 |
| Z12 | 2 | 1 | 16 |
| Z19 | 2 | 1 | 16 |
| Z22 | 1 | 0.5 | 17 |
| Z28 | 2 | 1 | 16 |
| Z31 | 1 | 1 | 17 |
| Z32 | 2 | 1 | 16 |
| Z34 | 1 | 0.75 | 17 |
| Z37 | 1 | 1 | 17 |
| Z38 | 2 | 1 | 16 |
| Z39 | 1 | 1 | 17 |
| Z41 | 1 | 0.75 | 16 |
| Z42 | 1 | 0.75 | 16 |
| Z48 | 0.5 | 1 | 17 |
| Z52 | 2 | 1 | 16 |
| Z54 | 0.5 | 1 | 17 |

BMD, broth microdilution；MTS, MIC Test Strip; DD, disk diffusion
