# Supplementary material for: Comparison of ceftobiprole 5 μg disk diffusion, MIC test strip, and broth microdilution for susceptibility testing of Staphylococcus aureus clinical isolates
Source: J Clin Microbiol. 2026 Mar 23;64(4):e00125-26. doi: 10.1128/jcm.00125-26 (PMC13059733; doi:10.1128/jcm.00125-26)
Supplement: Table S2 — Correlation among disk diffusion, MIC test strip, and BMD for MRSA isolates with ceftobiprole MTS of 2 mg/L. [file jcm.00125-26-s0002.doc]

Table S2. The correlation among disk diffusion, MTS, and BMD for MRSA with ceftobiprole MTS of 2mg/L

| Strain | BMD (mg/L) | MTS (mg/L) | DD (mm) |
| --- | --- | --- | --- |
| 408 | 0.5 | 2 | 17 |
| 462 | 1 | 2 | 17 |
| T16 | 2 | 2 | 13 |
| T18 | 2 | 2 | 12 |
| T33 | 1 | 2 | 13 |
| T42 | 0.5 | 2 | 17 |
| T71 | 2 | 2 | 14 |
| T114 | 1 | 2 | 15 |
| T168 | 2 | 2 | 13 |
| Z35 | 2 | 2 | 13 |

BMD, broth microdilution；MTS, MIC Test Strip; DD, disk diffusion
